# Assessment of community vulnerability and medical surge capacity in a foreseeable major disaster

**DOI:** 10.1371/journal.pone.0235425

**Published:** 2020-07-02

**Authors:** Soichiro Kato, Yoshihiro Yamaguchi, Ichiro Kawachi

**Affiliations:** 1 Department of Social and Behavioral Sciences, Harvard T.H. Chan School of Public Health, Boston, Massachusetts, United States of America; 2 Department of Trauma and Critical Care Medicine, Kyorin University School of Medicine, Mitaka, Tokyo, Japan; Pablo de Olavide University, SPAIN

## Abstract

**Objective:**

Developing an adequate disaster response capacity involves an assessment of available resources in areas that are vulnerable to disaster. Here, we sought to evaluate the gap between predicted damage in a foreseeable major earthquake versus existing municipality-level resources in Tokyo, Japan.

**Methods:**

Our study focused on the 53 municipalities in Tokyo to evaluate the relationships between the predicted number of severe casualties per 1,000 population from a future earthquake, community characteristics, and inpatient bed supply in local hospitals. Correlation analysis and supply–demand balance estimations were carried out at the municipality level, and the results were geographically visualized using choropleth maps.

**Results:**

The correlation analysis showed that higher casualties were correlated with municipalities with faster population increase, higher taxable incomes, lower unemployment rates, and higher bed volumes in disaster base hospitals. Under a maximal damage scenario in a future earthquake, we predict a shortage of 2,780 beds for the treatment of severe casualties across Tokyo. Even under a scenario of cooperation among neighboring municipalities, a shortage of 7,107 beds would remain.

**Conclusions:**

Tokyo is located in a zone where major earthquake damage is anticipated. Cooperation between neighboring municipalities may not suffice to address the undersupply of beds during the acute phase of a disaster. Hence, existing disaster preparedness plans require further reinforcement with a focus on local vulnerabilities.

## Introduction

Tokyo, Japan, is located on major fault lines, and a devastating near-field earthquake in Tokyo Bay is expected to hit the metropolitan area with overwhelming probability sometime in the future. In 2012, the Tokyo Metropolitan Government reported that the next catastrophic earthquake may occur within 30 years with a probability of 70% near the Tokyo metropolitan area [1]. The history and frequency of large-scale earthquakes suggest a high potential for the next disasters to occur in the near future. The government also estimated damages caused by the disaster with several patterns of earthquake types based on the latest models created by a special project [1]. Based on these patterns of damage predictions, an earthquake with an epicenter in the northern part of Tokyo Bay is expected to cause substantial damage, especially in the Tokyo metropolitan area [1].

In order to establish an adequate disaster response capacity in each community, efficient customization of resources and prioritization of subjects tailored to community-specific vulnerabilities are essential. A great deal of information about the relationships between the scale of damages in previous disasters and social factors at the community level has already been published [2–5]. Additionally, hospital surge capacity for critically ill and pediatric patients injured in disasters has been assessed on the basis of geographic information [6–9]. Several studies have reported associations among predicted casualties in a foreseeable disaster, area characteristics, and hospital surge capacities at the community level in a city [10–12]. Herein, we focus on identifying municipality-level vulnerabilities by utilizing forecast data for casualties in a future earthquake in a populated and coastal city in Japan.

In the aftermath of recent disasters in Japan, many hospitals at the affected sites experienced functional incapacitation from being completely or partially destroyed [13–17]. The loss of medical resources led to a greater concentration of critically ill and injured patients at the remaining facilities in the affected areas, underscoring the importance of the physical robustness of facilities as well as the local surge capacity for disaster medical preparedness. In the past two decades, the Japanese Ministry of Health, Labour and Welfare, and local governments have encouraged hospital structural strengthening and surge capacity securement centered on certified disaster base hospitals (DBHs), the Japan-specific term for regional hub hospitals focused on disaster response, in the medical districts of each prefecture [15,18]. Furthermore, various types of supportive operation systems centered around the DBHs, such as Disaster Medical Assistance Team (DMAT) deployment and acute-phase medical care teams, have been established [15–19]. To further optimize the preparedness for future events, the creation of data on the demand–supply balance between the predicted casualty volume and available medical resources can support the decisions of stakeholders regarding the preparation of medical services. In addition, identifying correlations between predicted casualties and community characteristics may be important for understanding the current situation and challenges in each community.

In this study, we analyzed the correlation between the predicted number of severe casualties per 1,000 population in each municipality and community factors, such as demographics and socio-economic factors, in Tokyo. Moreover, we estimated the supply–demand balance between severe casualties and the utilizable bed resources at DBHs to optimize resource placement and enhance cooperation among healthcare facilities.

## Materials and methods

### Study design

We evaluated the relationships between predicted disaster damages, community characteristics, and disaster medical preparedness at the municipality level in an urban area. We used data from a damage-prediction model for an earthquake affecting the metropolitan area in Japan. We utilized a case predicted by the model of an earthquake with a depth of 20–35 km underground, magnitude 7.3, with its epicenter in the northern part of Tokyo Bay [1]. The government set up a scenario in which the earthquake was to occur at 6 pm in the winter season with a wind speed of 8 m per second in order to maximize damage assumption. Our study focused on the 53 municipalities in 12 medical districts in Tokyo. Islands far from the mainland were excluded. According to a well-established framework, disaster preparedness consists of four key elements to strengthen surge capacity: staff, stuff, structure, and system [20]. Among these, structure, which is defined as the hardware (including inpatient beds), tends to be the least elastic in terms of response capacity in the immediate phase following a disaster, and covering the shortfall through external resources poses a daunting challenge. Our study focused on the structural surge capacity to handle severe casualties in each municipality.

### Study setting

We collected data for the predicted casualty ratio estimated in a 2012 report by the Tokyo metropolitan government [1]. In the report, the building collapse number for each 250 m square mesh was computed on the basis of seismic intensity distribution, building structures, building age, etc. These data were used as the elements of a detailed prediction to estimate the casualties in each municipality. We utilized the data showing the most significant damages, which were calculated under the scenario of an early evening event during the winter season. We selected the number of severe casualties from all predictions in the report to take into account the resources for critically ill or injured patients. The number of deaths, estimated up to 9,641, was not included.

The demographic and socio-economic factors in each municipality were collected from the Statistical Observations of Municipalities, which is a publicly available annual report published by the Statistics Bureau, Ministry of Internal Affairs and Communications [21]. Financial data for each municipality were collected from the List of Key Financial Indicators of Local Governments published by the same Ministry [22]. Since census data are collected once every 5 years in Japan, we utilized the data for the population in 2015 and the same period for other data.

Information about each hospital was collected from the 2018 data published by the Tokyo metropolitan government’s Social Welfare and Public Health Bureau [23]. The disaster response plan in Japan requires affected site DBHs to make concerted efforts to accept severe casualties [24]. Following this principle, we counted only the number of inpatient beds at DBHs as the capacity for severe casualty response. Our study used publicly available data and was exempt from local ethics board review.

### Measurements and outcomes

With regard to the variables of municipality demographics, we adopted population, population change rate from 2010 to 2015, land area, population density, mean age, gender ratio, and number of households. The absolute unemployment rate and taxable income per taxpayer were included as socio-economic factors. The tax revenue and total revenue per resident population, financial strength index, and cost of living for each municipality were abstracted from municipality financial statements. The financial capability index, defined as the average ratio of the standard fiscal revenue to the standard fiscal expenditure in the last 3 years, represents the financial capability of a local government [25]. The Laspeyres index, calculated from the weighted average of the salary comparison between local government employees and national government employees, indicates the cost of living and is commonly utilized to compare the price level among different communities [26].

The number of DBHs and DBH inpatient beds represent a proxy for the medical resources available for severe casualties. We estimated the maximum number of casualties that DBHs in each municipality could handle on the basis of their present bed capacity and adjustment coefficients. First, the number of usable inpatient beds (UIBs) immediately after the occurrence of a disaster was calculated from the number of total inpatient beds (TIBs) and mean vacant inpatient bed rate (VIBR). The VIBR for all hospitals in Tokyo was 20.3% in 2018 [27]. The equation for UIB is given below.

UIB=TIB×VIBR (a)

Then, we accounted for the number of additional vacant beds that could be made available by coordinating discharges for inpatients who do not require continued inpatient care in a disaster situation. A previous survey at hospitals in the United States suggested that an average of 20% inpatients can be discharged immediately after disaster occurrence [28]. The consensus reached for the care of critically ill and injured patients also recommended a level of preparedness for a 20% increase in bed capacity, independently in each advanced care facility [9]. In 2017, A questionnaire study involving DBHs in the Tokyo metropolitan area demonstrated that the expandable bed capacity was >10% on average, excluding efforts for inpatient discharges [29]. Although the total volume of expandable bed capacity in DBHs is unknown, we think that based on the report and consensus, the expected percentage of surge capacity in our study can be set to 20%. In Japan, the ability to utilize additional resources may vary depending on the certificated hospital grade for emergency response. Different from the DBH certification, highly functional facilities that receive critically ill and injured patients 24/7 during peace time are certified as “emergency medical care centers” (EMCCs). Facilities with sufficient resources and experience for emergency response may be able to provide a higher rate of extended capacity in emergencies than others. Therefore, we adopted an additional 20% increase in the number of inpatient beds for EMCC-certified DBHs compared to non-certified DBHs. Based on this adjustment, the acceptable number of severe casualties (ASC) for each DBH was calculated as below.

ASC=UIB+TIB×0.2×EMCCindex (b)

EMCCindex:1.2forEMCC-certified,1.0fornon-certified

We set the correlation coefficient (CC), standard deviations (SDs), and 95% confidence interval (95% CI) between the predicted severe casualties per 1,000 population in a future Tokyo earthquake and community characteristics at the municipality level as the primary outcomes. Additionally, we estimated the balance between severe casualties and ASC in each municipality. There were no obvious outliers and missing variables in our dataset.

### Data analysis

First, we calculated the Pearson correlation coefficients to assess the correlations between predicted severe casualties and municipality characteristics. Municipalities in Tokyo are divided into two major regions on the basis of the level of city development: 23 designated boroughs in the eastern region and suburban municipalities in the western region. In the government report, municipalities in the eastern region are expected to have more severe damages compared with those in the western region in a foreseeable earthquake [1]. In consideration of these backgrounds, we performed correlation analyses of the data for all municipalities (Model 1) as well as analysis limited to the 23 designated boroughs (Model 2). The statistically significant factors correlated with severe casualties for Model 1 were geographically visualized using choropleth maps. To assess the effect of differences in the severity of casualties, we also performed a sensitivity analysis replacing projected severe casualties with the total number of casualties per 1,000 population. Finally, the supply–demand balance between severe casualties and the ASC for DBHs was estimated at the municipality level. Further, the supply–demand balance reflecting the cooperation among neighboring municipalities was assessed. We defined cooperation as the transportation of surplus casualties from undersupplied municipalities to acceptable neighboring municipalities. We hypothesized that cooperation may be more effective if a municipality has remaining ASC capacity after receiving all severe casualties within its area and has a neighboring undersupplied municipality. In this situation, we applied transportation priority with a principle of east–west and south–north direction to escape from the seismic center while avoiding overlaps (S1 Fig). These balances were also visualized with maps.

Statistical analyses were performed with JMP (version 12.2.0; SAS Institute Inc., Cary, NC). All geographical analyses were conducted with Tableau Desktop (version 10.4.2; Tableau Software, Inc., Seattle, WA) using the basic map, for which Mapbox (https://www.mapbox.com/about/maps/) and OpenStreetMap Contributors (http://www.openstreetmap.org/copyright) hold the copyright. The shapefile utilized for creating the choropleth maps were collected from the National Land Information Division, National Spatial Planning and Regional Policy Bureau, Ministry of Land, Infrastructure, Transport and Tourism of Japan [30].

## Results

### Basic statistics

There are 13,488,780 individuals and 6,687,697 households in 53 municipalities of the Tokyo metropolitan area (Table 1). Demographic data showed that the population substantially varies among municipalities, from a minimum of 2,200 to a maximum of 9,033,000. These municipalities had an average population density of 10,738.5 individuals per square kilometers and a population growth of 2.5% from 2010 to 2015. There were approximately 50% single-person households, 11.2% single elderly-person households, and 9.4% elderly-couple households, indicative of an urban community structure and an aging society. The absolute unemployment rate was 4.0% on average. There were 43,933 staffed inpatient beds in 82 DBHs. In terms of the number of beds per resident population, there was an average of 3.4 beds per 1,000 population in 53 municipalities. Prediction of human damages in a future earthquake showed a maximum of 21,891 severe casualties out of 147,611 casualties in Tokyo. These estimates exclude the predicted 9,641 deaths. The average severe casualties at the municipality level was 1.6 per 1,000 population. Municipalities in the eastern region, near the predicted epicenter on the northern Tokyo Bay, showed much higher severe casualties than did municipalities in the western region.

**Table 1:** Municipality-level distributions of demographics, socio-economic factors, medical resources, and predicted maximum casualties in a future earthquake.

Municipality variable (n = 53)	Total	Mean	SD†	Minimum‡	Maximum¶
**Demographics**					
Population [*1,000]	13,488.8	254.5	214.7	2.2	903.3
Population growth [%]	-	2.5	6.0	-13.6	24.0
Area [/km2]	1,779.0	33.6	41.3	6.4	225.5
Population density [1,000/km^2^]	-	10.7	6.0	0.0	22.4
Mean age [years]	-	45.4	3.0	42.3	58.9
Gender ratio [male %]	-	49.3	1.0	47.2	51.5
Households [*1,000]	6,687.7	126.1	108.0	0.8	463.6
Nuclear household rate [%]	-	51.0	9.4	31.5	69.2
Single person household rate [%]	-	43.1	10.7	19.0	64.7
Elderly single household rate [%]	-	11.2	2.1	8.5	19.9
Elderly couple household rate [%]	-	9.4	3.3	5.0	18.8
**Socio-economic factors**					
Absolute unemployment rate [%]	-	4.0	0.7	1.8	5.4
Taxable income per taxpayer [*1,000 Yen]	-	4,147.3	1,369.4	2634.6	10,232.2
Tax revenue per capita [*1,000 Yen]	-	149.3	53.4	69.3	298.5
Total revenue per capita [*1,000 Yen]		451.2	228.3	309.0	1,655.5
Laspeyres Index	-	99.1	1.6	96.5	104.0
Financial capability index	-	0.75	0.28	0.16	1.44
**Medical resources**					
Disaster base hospitals	82	1.5	1.5	0.0	7.0
Bed volume in disaster base hospitals	43,933	828.9	1,072.6	0.0	5,446.0
per 1,000 population	-	3.4	4.1	0.0	21.4
**Predicted maximum casualties in a foreseeable earthquake**					
Casualties, total	147,611	2,785.1	3,407.0	0.0	10,412.0
per 1,000 population	-	11.4	25.6	0.0	177.4
Casualties, severe	21,891	413.0	544.6	0.0	1,855.0
per 1,000 population	-	1.6	3.4	0.0	23.2

† SD, standard deviation

‡ “Minimum” indicate the lowest score of each factor among municipalities

¶ “Maximum” indicate the highest score of each factor among municipalities

### Correlation analysis

**Fig 1:**
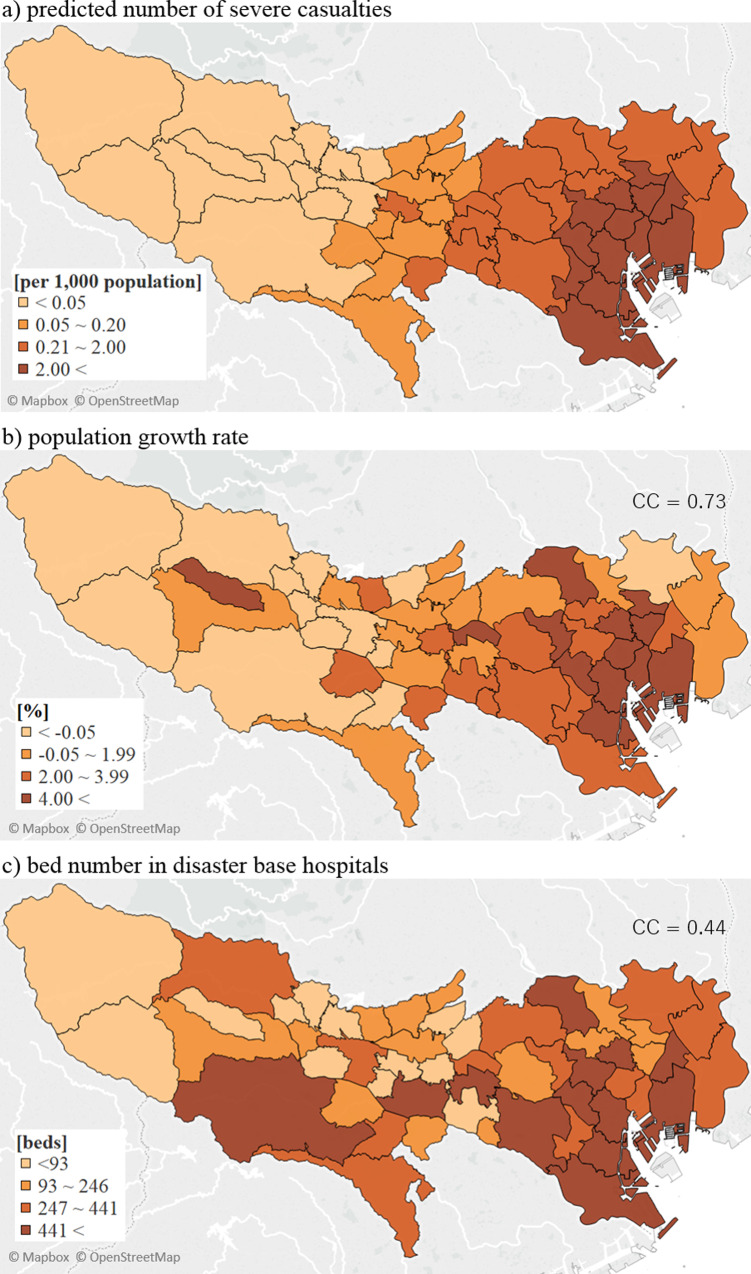
Geographic comparison of selected factors in correlation analysis: Choropleth maps. CC, correlation coefficient between the predicted number of severe casualties and each factor. Reprinted background map from Mapbox and OpenStreetMap under a CC BY license, with permission from Mapbox, original copyright 2020.

The distributions of predicted severe casualties and municipality characteristics in Tokyo are shown on choropleth maps (Fig 1), and the associations between these factors are represented by correlation coefficients (Table 2). The municipality factor that was most strongly correlated with severe casualties was the population growth rate (r = 0.73, 95% CI = 0.57 to 0.83). The single-person household prevalence also showed a statistically significant positive correlation (r = 0.45, 95% CI = 0.20 to 0.64) with severe casualties, whereas the elderly-couple household prevalence showed a negative correlation (r = −0.45, 95% CI = −0.64 to −0.21). Higher absolute unemployment rate in a municipality also correlated with lower casualties (r = −0.62, 95% CI = −0.77 to −0.43). The taxable income per taxpayer and tax revenue per capita showed a statistically significant correlation with severe casualties, r = 0.63 (95% CI = 0.44 to 0.77) and 0.28 (95% CI = 0.01 to 0.51), respectively. The Laspeyres index and financial strength index showed no significant association with casualties. The projected severe casualty rate and DBH beds per 1,000 population had a weak association (r = 0.44, 95% CI = 0.19 to 0.63), indicating that a higher bed capacity per population existed in places with higher expected damages. The sensitivity analysis, which utilized total casualties per 1,000 population (instead of severe casualties), indicated a similar pattern and grade of correlations. The population growth rate, absolute unemployment rate, and taxable income per taxpayer in Model 2 indicated similar patterns of correlation with the severe casualty rate compared with Model 1. The factors not correlated to severe casualties in Model 1, namely, the population density, total household number, mean age, and Laspeyres index, achieved statistical significance in Model 2. In contrast, the factors correlated with severe casualties in Model 1, such as the elderly couple household rate, single household rate, and DBH beds per 1,000 population, showed no significant correlations in Model 2.

**Table 2:** The correlation between municipality variables and predicted number of severe casualties in a foreseeable Tokyo earthquake.

Municipality variables	Correlation with predicted number of severe casualties #							
	Model 1				Model 2			
	Estimates		95% CI		Estimates		95% CI	
Population density [/km^2^]	0.17		-0.11	0.42	-0.60	**	-0.81	-0.25
Population growth rate [%]	0.73	***	0.57	0.83	0.78	***	0.55	0.90
Mean age [years]	-0.25		-0.48	0.03	-0.42	*	-0.71	-0.01
Gender rate (male) [%]	0.04		-0.23	0.31	0.11		-0.31	0.50
Households	0.04		-0.23	0.31	-0.53	**	-0.77	-0.15
Elderly couple household rate [%]	-0.45	***	-0.64	-0.21	-0.37		-0.68	0.05
Elderly single household rate [%]	-0.13		-0.39	0.15	-0.32		-0.65	0.10
Single household rate [%]	0.45	***	0.20	0.64	0.20		-0.23	0.57
Absolute unemployment rate [%]	-0.62	***	-0.76	-0.42	-0.70	***	-0.86	-0.40
Taxable income per taxpayer [1,000 Yen]	0.63	***	0.44	0.77	0.54	**	0.17	0.78
Tax revenue per capita [1,000 Yen]	0.28	*	0.01	0.51	0.68	***	0.38	0.85
Total revenue per capita [1,000 Yen]	0.29	*	0.02	0.52	0.93	***	0.84	0.97
Laspeyres index	-0.13		-0.38	0.15	0.44	*	0.03	0.72
Financial strengthen index	-0.13		-0.39	0.14	0.37		-0.05	0.68
Bed volume in disaster base hospitals #	0.44	**	0.19	0.63	0.40		-0.02	0.70

*, p < 0.05;

**, p < 0.01;

***, p < 0.001;

#, per 1,000 population

Model 1 included the data for all municipalities in Tokyo, except isolated islands. Model 2 included the data for only 23 designated boroughs in the eastern area of Tokyo.

### Supply–demand assessment

**Fig 2:**
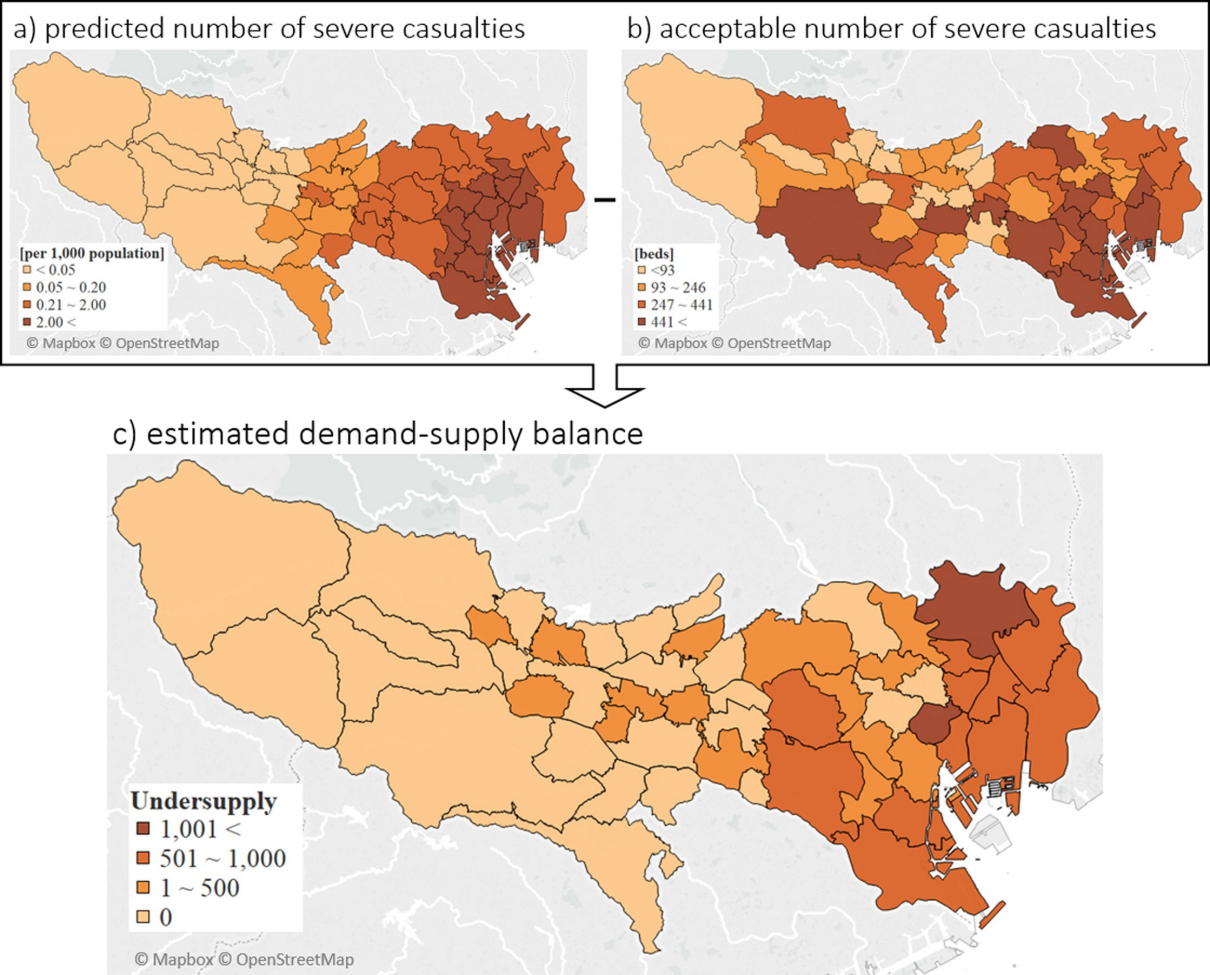
Geographic comparison between the predicted and acceptable number of severe casualties at the municipality level. Reprinted background map from Mapbox and OpenStreetMap under a CC BY license, with permission from Mapbox, original copyright 2020.

The total number of vacant inpatient beds in the whole city was estimated to be 10,954 in the immediate aftermath of an earthquake and ASC was estimated to be 19,111 (S1 Table). A simple calculation showed an absolute undersupply of bed resources by 2,780 for severe casualties in a future Tokyo Bay earthquake. We also visualized the supply–demand balance at the municipality level (Fig 2). The predicted casualties were concentrated in the southeastern municipalities near the seismic center and gradually decreased with the distance from the seismic center (Fig 2A). However, there was an inter-municipality disparity of ASC with no specific geographical trend (Fig 2B). Due to the disparity in distributions, large undersupplies were more common in the eastern coastal municipalities, especially in the northeastern area (Fig 2C). Some municipalities also showed undersupply despite having fewer severe casualties than others.

We also examined the capacity to meet the bed undersupply among neighboring municipalities. For each undersupplied municipality, we extracted and listed the name of neighboring bed-plenty municipalities, their capacity to receive patients from undersupplied municipalities, and the number of their neighboring undersupplied municipalities (S2 Table). For this simulation, we transferred the remaining severe casualties in undersupplied municipalities to neighboring bed-plenty municipalities by transferring severe casualties away from the seismic center (S1 Fig). The result showed a reduction of 4,187 severe casualties per 11,294 remaining casualties at undersupplied centers at the municipality level, which was less than half of the predicted undersupply.

## Discussion

In this study, some correlations were found between severe casualties in a foreseeable disaster and community characteristics in the Tokyo metropolitan area. Our projections imply an absolute supply shortage of inpatient bed capacity for severe casualties in a future large scale disaster. The capacity showed regional disparities that could not be resolved by only neighboring municipalities.

### Correlation between casualties and municipality characteristics

Although there is uncertainty surrounding the scale of future disasters, better response capability for foreseeable disasters may be fostered by a better understanding of the distributions or interplay of the community characteristics and hazards associated with human vulnerability. Clark et al. [12] listed the valuables of vulnerability that need to be addressed to assess the hazards and preparedness for a disaster: age, disabilities, family structure, income, race and ethnicity, etc. However, there have been no previous reports on the relationships between municipality characteristics and casualty distribution in Tokyo. Reports of relationships between predicted damages in foreseeable disasters and community characteristics are limited worldwide. The accumulation of data on many examples of vulnerability is necessary for further disaster preparedness development. Information on the correlation between disaster damages and community characteristics in Tokyo, a coastal city that is populated and rapidly aging, can serve as a useful reference not only for a data gathering community but also for worldwide communities that have similar social structures.

Our results from Model 1 showed correlations between the population growth rate, household structure rates, and severe casualties. Municipalities along the coastal area have a high concentration of these factors on the map (Fig 1). We interpreted this distribution to mean that the geographical characteristics of the eastern coastal area, near the predicted seismic center of a foreseeable earthquake with high damage risks, consist of continuous growth in the population size and relatively few elderly households. However, there was no correlation between casualties and household structures when we limited the analysis to the 23 designated boroughs. On the other hand, both Models 1 and 2 showed statistically significant correlations between low absolute unemployment rate, high taxable income per taxpayer, tax revenue per capita, and predicted severe casualties. These results indicate that the municipality with high severe casualty estimates presented high economic activity or high socio-economic status. Taking these results into account, once a large-scale disaster occurs, these high-risk municipalities are anticipated to have more significant human and economic damages than are other municipalities. Therefore, high-risk municipalities need to intensify their investment in disaster prevention and mitigation plans, including preparedness of medical surge capacity for casualties. The correlation between casualties and DBH inpatient bed capacity per 1,000 population showed a positive relationship among municipalities in Tokyo but was not statistically significant among the 23 designated boroughs. These results indicated that the inpatient bed surge capacity based on the predicted damages might not be sufficient at the municipality level in Tokyo, especially in areas that suffer more damages.

### Supply–demand balance

In the past two decades, communities in Japan have experienced many catastrophic events, including numerous deaths and injuries, such as the 1996 Hanshin-Awaji great earthquake, 2011 Great East Japan Earthquakes, and 2016 Kumamoto Earthquake [13–17]. As a result of these tragedies, Japanese governments and agencies have developed a disaster medical preparedness plan, including a system of DBHs, medical assistance teams, and communication networks [15,18,23,24]. Today, disaster medical care procedures for the acute phase have been established, consisting mainly of DBHs in the affected area [23]. The Tokyo metropolitan government has implemented a principle of hospital transportation prioritizing the transport of severe casualties to more DBHs than to non-DBHs [24]. The Ministry of Health, Labour, and Welfare recommended two times greater surge capacity for inpatient care at DBHs [15,18]. However, this was a comprehensive aim without specific numbers for the target. Generally, critically ill or injured patients require more medical equipment, resources, and staff per care than do mild to moderately ill patients. As already indicated in the methods section, previous reports noted that the margin of surge capacity in the acute phase is about 20% of the total bed amount. Few studies have been conducted to explore the community-wide medical surge capacity for severe casualties and the inter-municipality balance based on concrete and detailed damage prediction for foreseeable disasters in the world. Therefore, we implemented a supply–demand balance assessment of the severe casualty care for future disasters in order to reveal the present bed surge capacity depth in each municipality and disparities among its regions.

Less than half of the inpatient bed undersupply for severe casualties was covered by neighboring municipalities in our simulation, and the geographic visualization presented a random distribution for the remaining undersupply (Fig 2). These results indicate that the reconstruction and development of coalitions, transcending regional boundaries in some cases, are needed to ease the shortage in predicted undersupplied areas. In the United States, the Healthcare Coalition serving to strengthen intra-community cooperation for surge capacity securement was developed on top of individual capability enhancement [31]. It is also important in Japan to accelerate the establishment of a more flexible preparedness strategy with coalitions among municipalities or adjoining districts, which is at least partially pre-credentialed, based on in-depth damage, and supply prediction [16]. It is evident from past reports and our findings that the medical capabilities of DBHs in the affected areas must be reinforced in various ways to handle the remaining 2,000 severe casualties, such as by dispatching DMATs, external staff utilization, and hospital evacuation strategies [15–17,31–34]. Since substantial road collapse is expected in a foreseeable earthquake, the Tokyo metropolitan government has developed a plan for the restoration of major arterial roads by self-defense forces and other authorities [35]. Plans for transportation and evacuation by aircrafts have also been established; however, the transportation capacity will be limited in acute-phase disasters. In the past, a hospital ship from the US Navy was dispatched to affected islands after an earthquake and contributed to acute care, including surgical procedures [36]. Japan, which consists of narrow islands, is suitable for disaster response from offshore areas by ships when the infrastructure of the affected area is damaged, and this approach is already under consideration [37,38]. A comprehensive strategy that uses various methods is required.

### Limitations

We have some limitations with the internal and external validity in our study. First, human damages can change on the basis of the event type, scale of disaster, onset time, and weather conditions. We only considered one earthquake scenario from the government prediction model, and thus our analysis does not take into account the full range of alternative scenarios. However, the value of the simulation method in our study is to suggest a new line of approach for medical surge capacity assessment which could be adapted for alternative disaster scenarios.

ASC, a factor that we used to express the amount of acute medical resources for severe casualties, was calculated by adjusting the coefficient reflecting the facility certifications of hospitals in Japan. This variable might change depending on the significance of disaster damage and individual hospital preparedness for disasters. Even though the Japanese government has strengthened the equipment at DBHs to reinforce the capability to handle enormous damages following natural disasters, the problem of the submersion risk for DBHs near Tokyo Bay remains unresolved [18,23,24]. Once enormous damage occurs, the predicted capacities might be affected by various factors, potentially resulting in more or less capability for receiving and treating casualties.

Quantitative evaluations for the disaster response plan have yet to be unified. To facilitate an unbiased estimation, we took advantage of a specific response plan in Tokyo in which severe casualties may be transported preferentially to DBHs in their community. The types and sizes of disasters or the structure of disaster response plans differ depending on the region and country. Therefore, we are unable to generalize this original method to other communities. However, selecting the valuables of surge capacity evaluation may be useful for future quantitative analysis.

## Conclusions

In summary, our analysis suggested that severe casualties in a future major earthquake event is correlated with specific municipality characteristics and inpatient bed resources in Tokyo. In addition, we identified gaps between severe casualties and the capacity to handle casualties in DBHs among municipalities. To address this vulnerability, concrete steps for improving preparedness, such as narrowing inter-municipality gaps, specific procedures, and strengthening strategies for external resource loading, are needed. We hope that our study will contribute to better understandings of the current preparedness, and the procedure for demand-supply balance estimation might be applicable to other communities to assess the adequacy of medical surge capacity for critical events.

## Supporting information

S1 Fig The recommended direction of surplus severe casualty evacuation that minimizes undersupply without overlaps in a foreseeable earthquake. We attempted to offset the remaining severe casualties in undersupplied municipalities with the resources of neighboring municipalities while avoiding overlaps. The arrows indicate recommended directions of severe casualty evacuation based on a principle of east–west and south–north direction. The arrows are directed away from the seismic center except the case that it is unable to do so. The areas surrounded by lines are municipalities that have no neighboring bed-plenty municipalities in a disaster situation. Reprinted background map from Mapbox and OpenStreetMap under a CC BY license, with permission from Mapbox, original copyright 2020.
(TIF)

S1 Table The estimated acceptable number of severe casualties and bed-casualty balance in a foreseeable earthquake: At municipality level and secondary medical district level. URB, urban area; SUB, suburban area. † Weighted vacant bed number, (b), was calculated from vacant bed number in the immediate aftermath, (a), utilizing a weighting index whether the disaster base hospitals in the municipality was designated as the emergency medical care center or not. Both (a) and (b) were calculated by formulas which were indicated in the method section.
(DOCX)

S2 Table The estimated balance between bed-undersupplied municipalities and surrounding bed-plenty municipalities. † Municipality not neighboring any bed-plenty municipality in Tokyo, but neighboring municipality of other prefecture. ‡ Municipality not neighboring any bed-plenty municipality in Tokyo, but neighboring sea. ¶ In each line, we listed bed-plenty municipalities from left to right by giving priority to those on the western and northern sides of undersupplied municipalities. From left to right in each column, we listed the municipality name, available bed supply volume, and number of neighboring undersupplied municipalities. Bold letters indicated responsible municipalities that minimize undersupply volume while avoiding overlaps. § We calculated the remaining undersupplied bed volume among neighboring municipalities to add a dimension of overlapping needs. When an overlap of demand occurred in a supplying municipality, we prioritized a municipality closer to the seismic center.
(DOCX)
