# Supplementary figures and images for: Assessment of community vulnerability and medical surge capacity in a foreseeable major disaster
Source: PLoS One. 2020 Jul 2;15(7):e0235425. doi: 10.1371/journal.pone.0235425 (PMC7332042; doi:10.1371/journal.pone.0235425)

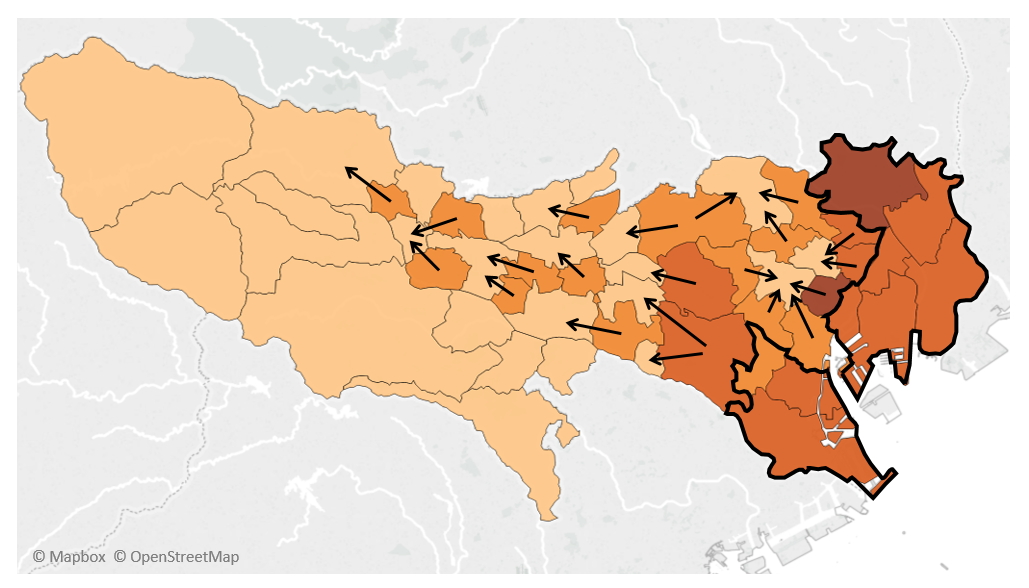

Supplement: S1 Fig — We attempted to offset the remaining severe casualties in undersupplied municipalities with the resources of neighboring municipalities while avoiding overlaps. The arrows indicate recommended directions of severe casualty evacuation based on a principle of east–west and south–north direction. The arrows are directed away from the seismic center except the case that it is unable to do so. The areas surrounded by lines are municipalities that have no neighboring bed-plenty municipalities in a disaster situation. Reprinted background map from Mapbox and OpenStreetMap under a CC BY license, with permission from Mapbox, original copyright 2020. (TIF) [file pone.0235425.s001.tif]
