# Supplementary material for: Assessment of community vulnerability and medical surge capacity in a foreseeable major disaster
Source: PLoS One. 2020 Jul 2;15(7):e0235425. doi: 10.1371/journal.pone.0235425 (PMC7332042; doi:10.1371/journal.pone.0235425)
Supplement: S1 Table — URB, urban area; SUB, suburban area. † Weighted vacant bed number, (b), was calculated from vacant bed number in the immediate aftermath, (a), utilizing a weighting index whether the disaster base hospitals in the municipality was designated as the emergency medical care center or not. Both (a) and (b) were calculated by formulas which were indicated in the method section. (DOCX) [file pone.0235425.s002.docx]

**S1 Table.** **The estimated acceptable number of severe casualties and bed-casualty balance in a foreseeable earthquake: at municipality level and secondary medical district level.**

| **Medical districts and municipalities** | **Estimated acceptable number  of severe casualties** † | | **Predicted severe casualties (c)** | **(b) - (c)** |
| --- | --- | --- | --- | --- |
|  | **Immediate aftermath (a)** | **Weighted (b)** |  |  |
| **Total** | **10954** | **19111** | **21891** | **-2780** |
| **Central, URB** | **2086** | **3673** | **5072** | **-1399** |
| Chiyoda | 200 | 345 | 1355 | -1010 |
| Chuo | 130 | 249 | 1023 | -774 |
| Minato | 483 | 815 | 1162 | -347 |
| Bunkyo | 1173 | 2104 | 608 | 1496 |
| Taito | 100 | 160 | 924 | -764 |
|  | **1004** | **1746** | **3231** | **-1485** |
| Shinagawa | 359 | 640 | 1376 | -736 |
| Ota | 645 | 1106 | 1855 | -749 |
| **Southwestern, URB** | **884** | **1566** | **2632** | **-1066** |
| Meguro | 190 | 364 | 576 | -212 |
| Setagaya | 400 | 639 | 1366 | -727 |
| Shibuya | 294 | 563 | 690 | -127 |
| **Western, URB** | **1684** | **2946** | **2138** | **808** |
| Shinjyuku | 1359 | 2427 | 887 | 1540 |
| Nakano | 177 | 283 | 356 | -73 |
| Suginami | 148 | 236 | 895 | -659 |
| **Northwestern, URB** | **1176** | **2047** | **1188** | **859** |
| Toshima | 127 | 203 | 279 | -76 |
| Kita | 85 | 136 | 268 | -132 |
| Itabashi | 779 | 1412 | 226 | 1186 |
| Nerima | 185 | 296 | 415 | -119 |
| **Northeastern, URB** | **513** | **856** | **2899** | **-2043** |
| Arakawa | 112 | 214 | 753 | -539 |
| Adachi | 182 | 292 | 1294 | -1002 |
| Katushika | 219 | 350 | 852 | -502 |
| **Eastern, URB** | **933** | **1553** | **4175** | **-2622** |
| Sumida | 241 | 446 | 1312 | -866 |
| Koto | 420 | 672 | 1654 | -982 |
| Edogawa | 272 | 435 | 1209 | -774 |
| **Western, SUB** | **295** | **515** | **4** | **511** |
| Oume | 140 | 268 | 0 | 268 |
| Fussa | 79 | 126 | 1 | 125 |
| Hamura | 0 | 0 | 1 | -1 |
| Akiruno | 76 | 121 | 2 | 119 |
| Mizuho | 0 | 0 | 0 | 0 |
| Hinode | 0 | 0 | 0 | 0 |
| Hinohara | 0 | 0 | 0 | 0 |
| Okutama | 0 | 0 | 0 | 0 |
| **Southern, SUB** | **761** | **1298** | **142** | **1156** |
| Hachioji | 277 | 491 | 19 | 472 |
| Machida | 166 | 266 | 76 | 190 |
| Hino | 75 | 120 | 13 | 107 |
| Tama | 171 | 306 | 16 | 290 |
| Inagi | 72 | 115 | 18 | 97 |
| **West of Northern, SUB** | **296** | **509** | **49** | **460** |
| Tachikawa | 225 | 396 | 7 | 389 |
| Akishima | 0 | 0 | 2 | -2 |
| Kokubunji | 0 | 0 | 31 | -31 |
| Kunitachi | 0 | 0 | 4 | -4 |
| Higashiyamato | 71 | 113 | 4 | 109 |
| Musashimurayama | 0 | 0 | 1 | -1 |
| **South of Northern, SUB** | **922** | **1722** | **271** | **1451** |
| Musashino | 152 | 291 | 83 | 208 |
| Mitaka | 288 | 553 | 80 | 473 |
| Fuchu | 337 | 646 | 15 | 631 |
| Chofu | 0 | 0 | 51 | -51 |
| Koganei | 0 | 0 | 22 | -22 |
| Komae | 145 | 232 | 20 | 212 |
| **North of Northern, SUB** | **400** | **680** | **90** | **590** |
| Kodaira | 129 | 247 | 17 | 230 |
| Higashimurayama | 86 | 137 | 18 | 119 |
| Kiyose | 140 | 224 | 5 | 219 |
| Higashikurume | 0 | 0 | 12 | -12 |
| Nishitokyo | 45 | 72 | 38 | 34 |
