# Supplementary material for: Assessment of community vulnerability and medical surge capacity in a foreseeable major disaster
Source: PLoS One. 2020 Jul 2;15(7):e0235425. doi: 10.1371/journal.pone.0235425 (PMC7332042; doi:10.1371/journal.pone.0235425)
Supplement: S2 Table — † Municipality not neighboring any bed-plenty municipality in Tokyo, but neighboring municipality of other prefecture. ‡ Municipality not neighboring any bed-plenty municipality in Tokyo, but neighboring sea. ¶ In each line, we listed bed-plenty municipalities from left to right by giving priority to those on the western and northern sides of undersupplied municipalities. From left to right in each column, we listed the municipality name, available bed supply volume, and number of neighboring undersupplied municipalities. Bold letters indicated responsible municipalities that minimize undersupply volume while avoiding overlaps. § We calculated the remaining undersupplied bed volume among neighboring municipalities to add a dimension of overlapping needs. When an overlap of demand occurred in a supplying municipality, we prioritized a municipality closer to the seismic center. (DOCX) [file pone.0235425.s003.docx]

**S2 Table. The estimated balance between bed-undersupplied municipalities and surrounding bed-plenty municipalities.**

| **Undersupplied municipalities** | | **Plenty municipalities neighboring each undersupplied municipality** ¶ | | | | | **Remaining shortage** § **(-7,107)** |
| --- | --- | --- | --- | --- | --- | --- | --- |
| **Name** | **shortage  (-11,294)** | **1** | **2** | **3** | **4** | **5** |  |
| **Central, URB** |  |  |  |  |  |  |  |
| Chiyoda | -1,010 | **Sh, 1,540, 5** | Bu, 1,496, 5 |  |  |  | 0 |
| Chuo | -774 | ‡ |  |  |  |  | -774 |
| Minato | -347 | **Sh, 1,540, 5** |  |  |  |  | 0 |
| Taito | -764 | **Bu, 1,496, 5** |  |  |  |  | 0 |
| **Southern, URB** |  |  |  |  |  |  |  |
| Shinagawa | -736 | ‡ |  |  |  |  | -736 |
| Ota | -749 | †‡ |  |  |  |  | -749 |
| **Southwestern, URB** | |  |  |  |  |  |  |
| Meguro | -212 |  |  |  |  |  | -212 |
| Setagaya | -727 | **Mi, 473, 4** | **Km, 212, 2** |  |  |  | -42 |
| Shibuya | -127 | **Sh, 1,540, 5** |  |  |  |  | 0 |
| **Western, URB** |  |  |  |  |  |  |  |
| Nakano | -73 | **Sh, 1,540, 5** |  |  |  |  | -17 |
| Suginami | -659 | **Mu, 208, 3** | Mi, 473, 4 |  |  |  | -451 |
| **Northwestern, URB** | |  |  |  |  |  |  |
| Toshima | -76 | **It, 1,186, 3** | Sh, 1,540, 5 | Bu, 1,496, 5 |  |  | 0 |
| Kita | -132 | **It, 1,186, 3** | Bu, 1,496, 5 |  |  |  | 0 |
| Nerima | -119 | **Ni, 34, 3** | Mu, 208, 3 | **It, 1,186, 3** |  |  | 0 |
| **Northeastern, URB** |  |  |  |  |  |  |  |
| Arakawa | -539 | **Bu, 1,496, 5** |  |  |  |  | 0 |
| Adachi | -1,002 | † |  |  |  |  | -1,002 |
| Katushika | -502 | † |  |  |  |  | -502 |
| **Eastern, URB** |  |  |  |  |  |  |  |
| Sumida | -866 |  |  |  |  |  | -866 |
| Koto | -982 | † |  |  |  |  | -982 |
| Edogawa | -774 | †‡ |  |  |  |  | -774 |
| **Western, SUB** |  |  |  |  |  |  |  |
| Hamura | -1 | **Ou, 268** | Ak, 119 | Fs, 125, 3 |  |  | 0 |
| **West of Northern, SUB** | |  |  |  |  |  |  |
| Akishima | -2 | **Fs, 125, 3** | Ha, 472 | Ta, 389, 4 | Hn, 107, 2 |  | 0 |
| Kokubunji | -31 | **Ta, 389, 4** | Ko, 230, 3 | Fc, 631, 4 |  |  | 0 |
| Kunitachi | -4 | **Ta, 389, 4** | Hn, 107, 2 | Fc, 631, 4 |  |  | 0 |
| Musashimurayama | -1 | **Fs, 125, 3** | Hy, 109 | Ta, 389, 4 |  |  | 0 |
| **South of Northern, SUB** | |  |  |  |  |  |  |
| Chofu | -51 | **Fc, 631, 4** | In, 97 | Mi, 473, 4 | Km, 212, 2 |  | 0 |
| Koganei | -22 | **Ko, 230, 3** | Ni, 34, 3 | Mu, 208, 3 | Fc, 631, 4 | Mi, 473, 4 | 0 |
| **North of Northern, SUB** | |  |  |  |  |  |  |
| Higashikurume | -12 | **Hm, 119** | Ki, 219 | Ko, 230, 3 | Ni, 34, 3 |  | 0 |
